# Endothelial-derived complement factor D contributes to endothelial dysfunction in malignant nephrosclerosis via local complement activation

**DOI:** 10.1038/s41440-023-01300-3

**Published:** 2023-05-15

**Authors:** Zheng Wang, Zhe Zhang, Yuan Li, Ying Zhang, Min Wei, Hui Li, Shanzhi Yang, Yali Zhou, Xinjin Zhou, Guolan Xing

**Affiliations:** 1 Department of Nephrology, First Affiliated Hospital of Zhengzhou University, Zhengzhou, Henan People’s Republic of China; 2 Academy of Medical Sciences, Zhengzhou University, Zhengzhou, Henan People’s Republic of China; 3 Department of Pathology, Baylor University Medical Center at Dallas, Dallas, TX USA

**Keywords:** Malignant nephrosclerosis, Endothelial cells, Local complement, Alternative pathway, Complement factor D

## Abstract

Malignant nephrosclerosis is a thrombotic microangiopathy associated with abnormal local activation of the complement alternative pathway (AP). However, the mechanism underlying local AP activation is not fully understood. We hypothesized that complement factor D (CFD) secreted by endothelial cells triggers vascular dysfunction in malignant nephrosclerosis via local complement activation. We investigated the deposition of CFD in human kidney biopsy tissues and the function of endothelial-derived CFD in endothelial cell cultures. Immunofluorescence microscopy and laser microdissection-targeted mass spectrometry revealed significant deposition of CFD in the kidneys of patients with malignant nephrosclerosis. Conditionally immortalized human glomerular endothelial cells (CiGEnCs) continuously expressed and secreted CFD in vitro. CFD knockdown in CiGEnCs by small interfering RNA reduced local complement activation and attenuated the upregulation of intercellular adhesion molecule-1 (ICAM-1), vascular adhesion molecule-1 (VCAM-1), von Willebrand factor (VWF), and endothelin-1 (ET-1) induced by Ang II. The expression of CFD in CiGEnCs was significantly higher than that in other types of microvascular endothelial cells. Our findings suggest that (i) glomerular endothelial cells are an important source of local renal CFD, (ii) endothelial-derived CFD can activate the local complement system, and (iii) endothelial-derived CFD mediates endothelial dysfunction, which may play a role in the pathogenesis of malignant nephrosclerosis.

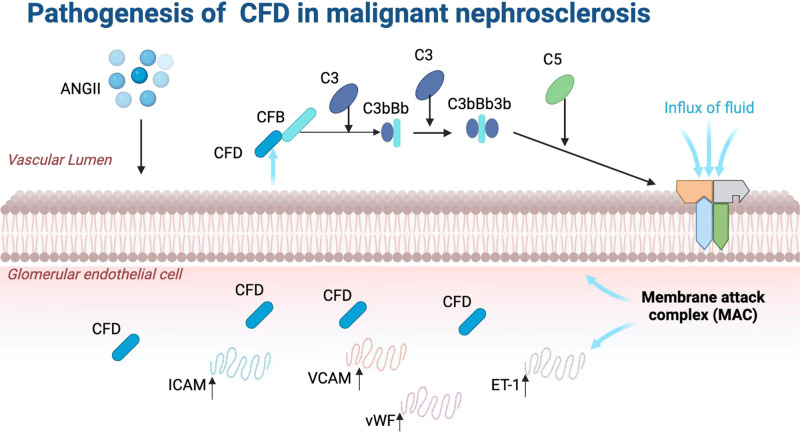

## Introduction

Malignant nephrosclerosis, triggered by severe hypertension (HTN), is a clinical emergency, in which the kidney may disclose features of thrombotic microangiopathy [1]. The mechanism of malignant nephrosclerosis is not completely understood, and deterioration in renal function cannot be attributed solely to accelerated HTN [2]. In some patients with malignant nephrosclerosis, renal impairment continues to progress to end-stage renal disease even when blood pressure is well controlled [3]. The effective and targeted prevention and treatment of malignant nephrosclerosis depend on a better understanding of its pathogenesis.

The complement system is an important part of the innate immunity and plays a critical role in clearing pathogens through three pathways: the classical, lectin, and alternative pathways [4–8]. However, complement activation is a double-edged sword, and abnormal activation of the complement system can lead to endothelial dysfunction [9]. Various studies have suggested a role of alternative pathway (AP) in the development of malignant nephrosclerosis [1, 10–13]. Malignant nephrosclerosis is histologically similar to atypical hemolytic uremic syndrome (aHUS), a disease that has been shown to be curable by complement inhibition therapy using the C5 cleavage inhibitor eculizumab, and is presented with features such as complement deposition, inflammation, and thrombotic microangiopathy [14, 15]. Vascular endothelial dysfunction caused by the membrane attack complex (MAC), the end product of complement activation, promotes the progression of malignant nephrosclerosis [8, 16].

Our previous study showed that local activation of the AP in the kidneys may also be involved in the pathogenesis of malignant nephrosclerosis as does systemic circulation. Although elevated plasma and urinary levels of complement AP components (factor D, factor B, properdin, C3a, and C5a) were observed in patients with malignant nephrosclerosis compared with normal controls, urinary but not plasma levels of AP-related complement components were correlated with renal function, prognosis, and pathological changes [1]. The mechanism underlying local activation of the complement system has not been elucidated.

Although complements were initially thought to be derived from the liver, most cell types can synthesize complements, and the functional importance of complements from extrahepatic sources has long been established [17]. Endothelial cells, the inner cells of blood and lymphatic vessels, have been found to express various complement components [18, 19]. It has been reported that abnormally increased complement factor D (CFD) in the microparticles of endothelial cells in patients with chronic kidney disease (CKD) may play a role in CKD-related vascular disease [20]. CFD, the rate-limiting enzyme in the activation of the alternative complement pathway, drives the alternative complement cascade by binding to and cleaving complement factor B, and increased local levels of CFD can trigger aberrant activation of the complement system [21–24]. Therefore, we hypothesized that local complement regulation by endothelial cells via CFD production and secretion may play a role in malignant nephrosclerosis.

## Materials and methods

### Patients

Five patients with malignant nephrosclerosis diagnosed through renal biopsy in the Department of Nephrology of the First Affiliated Hospital of Zhengzhou University from January 2021 to December 2021 were enrolled in this study. In addition, three cases of zero-hour allograft biopsies during the corresponding period were selected as negative controls. Ethical approval was obtained from the Ethics Committee of Zhengzhou University. The baseline demographic, clinical, and laboratory data of the patients are shown in Table S1. Informed consent was obtained for renal biopsy from each patient.

### Laser microdissection-targeted mass spectrometry for quantification of the unique peptides of CFD and C5b-9 (C8a, C8b, and C9)

The glomeruli and arterioles of renal tissues from patients with malignant nephrosclerosis and the negative control group were obtained by laser microdissection (LMD7; Leica AG, Heerbrugg, Switzerland) of a total area of approximately 30,0000 μm^2^. After ultrasonic crushing, trypsin digestion, and desalination, the samples were loaded onto a Q-Exactive HF-X mass spectrometer (Thermo Fisher Scientific GmbH, Bremen, Germany) and quantitatively analyzed by parallel reaction monitoring (PRM). We analyzed the unique peptides of CFD and C5b-9 (C8a, C8b, and C9) normalized to the sum of the full MS1 scans for relative quantification. The unique peptides we selected included RPDSLQHVLLPVLDR, ATLGPAVRPLPWQR, and VQVLLGAHSLSQPEPSK from CFD (UniProt ID: P00746), LYYGDDEK from C8a (UniProt ID: P07357), LPLEYSYGEYR from C8b (UniProt ID: P07358), and ALPTTYEK and LSPIYNLVPVK from C9 (UniProt ID: P02748). Raw files obtained from PRM acquisitions were analyzed using the Skyline software (version 3.7) (http://proteome.gs.washington.edu/software/skyline). The MS spectrum of each peptide was reconstructed from the area under the curve (AUC), and the AUC for each peptide was obtained by adding the AUCs of the corresponding common co-eluted fragment ions.

### Cell culture and treatment

Conditionally immortalized human glomerular endothelial cells (CiGEnCs) and human microvascular endothelial cells (HMECs) of dermal origin were used in our study. HMECs (HMEC-1; CRL3243) were purchased from the ATCC. CiGEnCs isolated using a standard protocol [25] were donated by Shandong University. The complete culture medium for HMECs and CiGEnCs was endothelial cell medium (ScienCell Research Laboratories, Carlsbad, CA, USA) supplemented with endothelial cell growth factor, antibiotics (100 U/mL penicillin/100 μg/mL streptomycin), and 10% fetal bovine serum. Cells were cultured in humidified air with 5% CO_2_ at 37 °C and were used below passage 10 when they were >80% confluent. Ang II (MedChemExpress, Monmouth Junction, NJ, USA) diluted in the medium was added to the cell culture plates for 12–24 h. The fresh sera of healthy donors were centrifuged at 12,000 rpm at 4 °C for 5 min and frozen at –80 °C until required. the cells were incubated with 5 μl serum at 37 °C for 35 min.

### Cell viability assay

Cell viability assay was performed using Cell Counting Kit-8 (CCK-8) (Dojindo, Kumamoto, Japan). The cells were seeded into four separate 96-well plates, two of which were used to assess stimulation by Ang II at a concentration of 0.1 μM, and the other two were used at a concentration of 1 μM. After the cells were incubated with each concentration of Ang II for 12 h or 24 h, the CCK-8 solution was diluted in the medium and added to each 96-well plate. The absorbance of each well was measured using a microplate reader.

### RNA interference

RNA interference was performed using small interfering RNAs (siRNAs) specifically targeting CFD (100 nM final concentration) (GenePharma, Shanghai, China). The sense RNA sequence was 5ʹ-GCAUCUGGUUGGUCUUUAUTT-3ʹ, and the antisense RNA sequence was 5ʹ-GCGTTTGTTTAGTTCACTTGT-3ʹ. A negative siRNA control (GenePharma, Shanghai, China) was used as the negative control. The sense RNA sequence was 5ʹ-UUCUCCGAACGUGUCACGUTT-3ʹ, and the antisense RNA sequence was 5ʹ-ACGUGACACGUUCGGAGAATT-3ʹ. Cell cultures with 80–90% confluence were transiently transfected with selected siRNAs using Lipofectamine 3000 (Invitrogen, Life Technologies, Carlsbad, CA, USA), following the manufacturer’s instructions. After 6 h of cell incubation with the transfection cocktail, CiGEnCs were incubated in fresh growth medium for 24 h. Western blot and real-time PCR (qPCR) assays were performed to assess the efficiency of siRNA transfection.

### qPCR validation

Total mRNA was extracted from unstimulated and Ang II-stimulated HMECs and CiGEnCs using TRIzol reagent (Thermo Fisher Scientific, MA, USA), and cDNA was synthesized using a reverse transcription kit (Takara, Japan). qPCR was performed using Taq Pro Universal SYBR qPCR Master Mix (Vazyme, Nanjing, China) according to the manufacturer’s instructions. The mRNA expression levels of all targets were normalized to the level of GAPDH, which was used as the loading control, and the results were analyzed using the 2^–ΔΔCt^ method. The qPCR primers that were used are listed in Table S2.

### Western blot analysis

The proteins of endothelial cells were obtained from lysates, which were prepared using RIPA buffer (Solarbio, Beijing, China) and PMSF (Solarbio, Beijing, China) at a ratio of 100:1. Supernatant was transferred to a fresh test tube and protein solutions were precipitated using acetone (1:4, protein solution-acetone) overnight at −20 °C. Following precipitation, samples were centrifuged (15,000 × *g*, 15 min, 4 °C) and pellets were air-dried to eliminate residual acetone. After quantifying their concentrations with the BCA Protein Assay Kit (Solarbio, Beijing, China), the proteins were boiled with 5× NuPAGE LDS Sample Buffer (Invitrogen, Life Technologies, Carlsbad, CA, USA). Protein samples (10 µg) were separated by 10% SDS-PAGE gels (Epizyme, Shanghai, China) and transferred to PVDF membranes (Millipore Sigma, Burlington, MA, USA). After blocking with 5% nonfat milk in 0.1% TBST for 2 h at room temperature, the membranes were incubated with primary antibodies at 4 °C overnight. Subsequently, the membranes were rinsed with 0.1% TBST three times and incubated with secondary antibodies for 2 h. The primary antibodies against CFD (catalog number: 26050-1-AP; Proteintech, Chicago, USA), von Willebrand factor (vWF, catalog number: 11778-1-AP; Proteintech, Chicago, USA), endothelin-1(ET-1, catalog number: 67008-1-Ig; Proteintech, Chicago, USA), vascular adhesion molecule-1 (VCAM-1, catalog number: 66294-1-Ig; Proteintech, Chicago, USA), and intercellular adhesion molecule-1 (ICAM-1, catalog number: 60299-1-Ig; Proteintech, Chicago, USA) were used at a dilution of 1:500–1,000. The secondary antibodies were HRP-conjugated Affinipure Goat Anti-Mouse IgG (catalog number: SA00001-1; Proteintech, Chicago, USA) and HRP-conjugated Affinipure Goat Anti-Rabbit IgG (catalog number: SA00001-2; Proteintech, Chicago, USA). β-actin (catalog number:ab8226;Abcam, Cambridge, UK) was used as an internal control and at a dilution of 1:1,000. Specific protein bands were detected using the Immobilon Western HRP Substrate (Millipore Sigma, Burlington, MA, USA) and quantified by ImageJ software.

### Immunofluorescence

Paraffin sections were dewaxed by soaking them in xylene with decreasing concentrations of alcohol. Antigen repair was performed by microwave heating in citrate buffer, followed by trypsin digestion at 37 °C for 10 min. CiGEnCs were cultured and treated on glass coverslips. After treatment, the cells were fixed with 4% paraformaldehyde for 20 min, permeabilized with 0.05% Triton-X 100 for 30 min, and blocked with 5% bovine serum albumin for 60 min at room temperature.

Paraffin sections and cells were incubated with primary antibodies at 4 °C overnight, followed by incubation with secondary antibodies for 2 h at room temperature. Subsequently, the sections and cells were counterstained with DAPI (5 μg/ml; Sigma-Aldrich) for 5 min and observed under a fluorescence microscope (Nikon, Tokyo, Japan). Antibodies against C5b-9 (catalog number: ab55811; Abcam, Cambridge, UK), CFD (catalog number: ab213682; Abcam, Cambridge, UK), and CD34 (catalog number: 60180-1-Ig; Proteintech, Chicago, USA) were used as primary antibodies. Goat Anti-Rabbit IgG(H + L)—FITC conjugate (catalog number: SA00003-2; Proteintech, Chicago, USA), Goat Anti-Rabbit IgG(H + L)—Cy3 conjugate (catalog number: SA00009-2; Proteintech, Chicago, USA), and Goat Anti-Mouse IgG(H + L)—FITC conjugate (catalog number: SA00003-2; Proteintech, Chicago, USA) were used as secondary antibodies. Images were obtained randomly and quantified using ImageJ software.

### Statistical analysis

All data are presented as the mean ± standard deviation (SD). Shapiro-Wilks tests were used to evaluate normal distribution of variables. the student’s t-test was used to compare different groups between mean values in two groups for unpaired samples and analysis of variance (ANOVA) was used in multiple comparison tests for normally distributed variables, Meanwhile, the Kruskal-Wallis test was used for nonnormally distributed variables. Statistical analysis of the data was performed using GraphPad Prism 6.0 (GraphPad Software Inc., USA). All experiments were repeated at least three times, and differences were considered significant when *p* was less than 0.05.

## Results

### Immunofluorescence and laser microdissection-targeted mass spectrometry of the deposition of CFD in the kidneys of patients with malignant nephrosclerosis

**Fig. 1:**
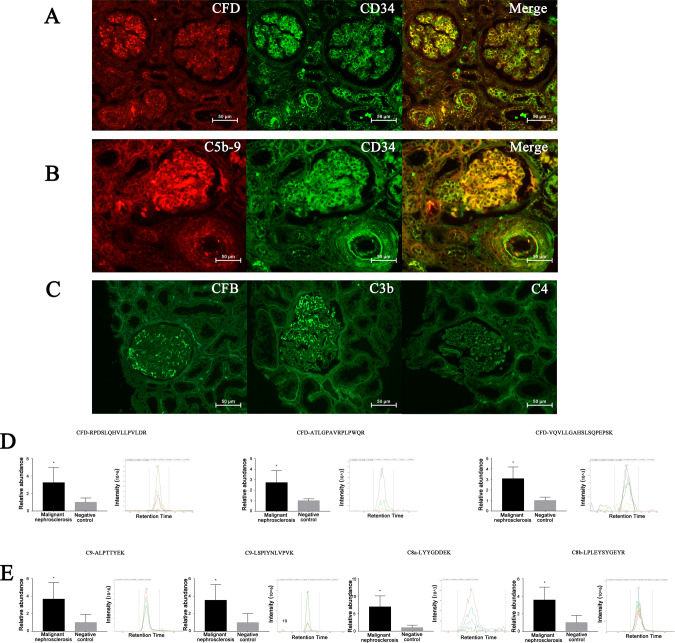
Immunofluorescence and targeted mass spectrometry quantification of renal specimens from patients with malignant nephrosclerosis. **A** IF microscopy showing the localization of CFD (red), CD34 (green), and DAPI (blue) and merged image showing the co-localization of CFD and CD34 (yellow). **B** IF microscopy showing the localization of C5b-9 (red), CD34 (green), and DAPI (blue) and merged image showing the co-localization of C5b-9 and CD34 (yellow). **C** IF microscopy showing the localization and Intensity of CFB, C3 and C4. **D** Area under the curve (AUC) of RPDSLQHVLLPVLDR, ATLGPAVRPLPWQR, and VQVLLGAHSLSQPEPSK (unique peptides of CFD) showing the relative abundance and spectra showing the co-eluted fragment ions of the corresponding peptides. **E** AUC of ALPTTYEK, LSPIYNLVPVK, LYYGDDEK, and LPLEYSYGEYR (unique peptides of C5b-9) (C8a, C8b, and C9) showing the relative abundance and spectra showing the co-eluted fragment ions of the corresponding peptides. All data are the mean ± SD; *n* ≥ 3 per group; *p < 0.05 compared with the expression in the negative control group. Scale bars represent 50 μm

In the renal biopsy specimens of patients with malignant nephrosclerosis, CFD deposits were detected along the walls of the arteries/arterioles and glomerular capillaries by immunofluorescence and colocalized with the endothelial cell marker CD34 (Fig. 1A). The abundance of unique peptides from CFD (RPDSLQHVLLPVLDR, ATLGPAVRPLPWQR, and VQVLLGAHSLSQPEPSK) in the glomeruli and arterioles of patients with malignant nephrosclerosis was increased significantly compared with that in the renal specimens of the negative controls (Fig. 1D).

Locally deposited CFD may play a role in the pathogenesis of malignant nephrosclerosis via local complement activation as locally increased CFD can activate the complement AP. The deposition of C5b-9 was observed along the walls of arteries/arterioles and glomerular capillaries, and the abundance of C5b-9 (C8a, C8b, and C9) unique peptides (ALPTTYEK, LSPIYNLVPVK, LYYGDDEK, and LPLEYSYGEYR) was significantly increased in the renal specimens of patients with malignant nephrosclerosis (Fig. 1B, E) when compared with normal controls. To examine whether AP activation has any influence on complement classical pathway and lectin pathway, we examined the expression of CFB, C3 and C4 by immunofluorescent staining. The data indicated that C3 was highly expressed in glomeruli endothelial cells, followed by CFB and C4 accumulation the lowest (Fig. 1C). Together, these data suggest that CFD might be responsible for triggering AP activation in malignant nephrosclerosis.

### Dysfunction of CiGEnCs following stimulation by Ang II in vitro

**Fig. 2:**
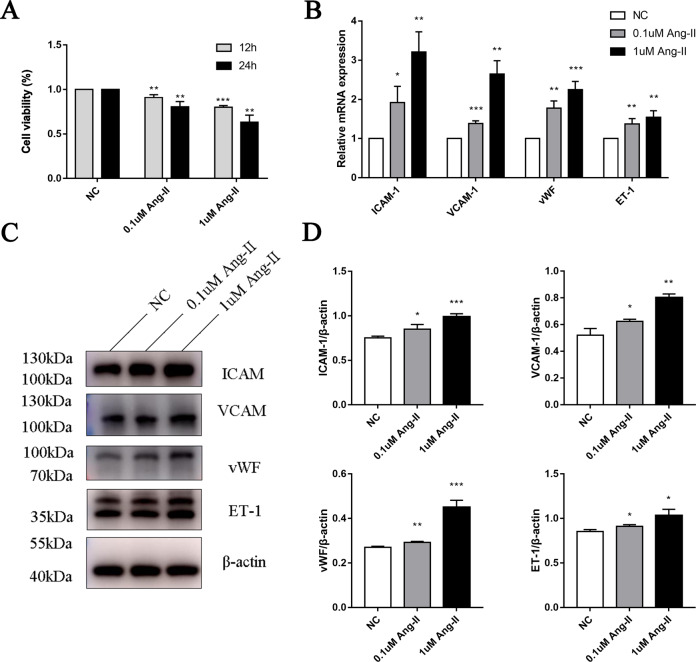
Cell viability of CiGEnCs and expression of ICAM-1, VCAM-1, vWF, and ET-1 in CiGEnCs stimulated with different concentrations of Ang II. **A** CCK-8 analysis of the cell viability of endothelial cells stimulated under different conditions. **B** mRNA expression of ICAM-1, VCAM-1, vWF, and ET-1 in endothelial cells incubated for 24 h with the negative control (white bars), 0.1 μm Ang II (gray bars), and 1 μm Ang II (black bars). **C**, **D** Protein expression of ICAM-1, VCAM-1, vWF, and ET-1 in cultured endothelial cells after stimulation with different concentrations of Ang II. All data are the mean ± SD; *n* ≥ 3 per group; *p < 0.05, **p < 0.01, ***p < 0.001 compared with the expression in the negative control group

CiGEnCs were cultured under Ang II stimulation to simulate the activated renin-angiotensin system in patients with malignant HTN. We assessed whether the in vitro injury model of CiGEnCs was successfully constructed under Ang II stimulation. CCK-8 assay was performed to determine the viability of CiGEnCs, and the results (Fig. 2A) showed that cell viability was decreased significantly after 24 h of Ang II stimulation. The expression of ICAM-1, VCAM-1, VWF, and ET-1 in glomerular endothelial cells, which reflects the degree of endothelial cell activation [26–32], was used to evaluate the degree of endothelial cell injury. As expected, with the enhancement of Ang II stimulation, both mRNA and protein expression levels of ICAM-1, VCAM-1, vWF, and ET-1 were significantly upregulated (Fig. 2B–D).

### Expression and secretion of CFD by CiGEnCs in vitro

**Fig. 3:**
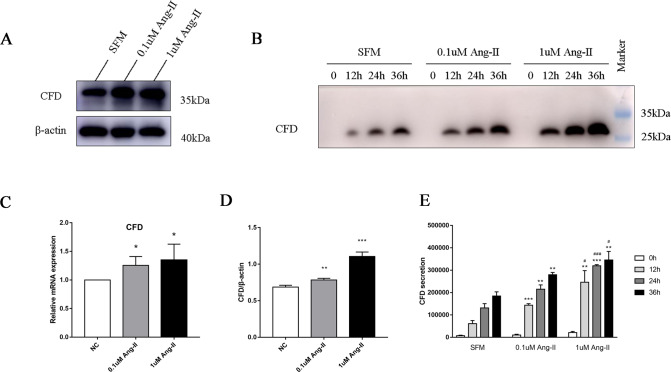
Expression and secretion of CFD by CiGEnCs. **A**, **D** Protein expression of CFD in CiGEnCs after stimulation with different concentrations of Ang II. **B** Secretion of CFD in the supernatant of CiGEnCs stimulated with different concentrations of Ang II for 12 h, 24 h, and 36 h. **C** mRNA expression of CFD in CiGEnCs incubated for 24 h with the negative control (white bars), 0.1 μm Ang II, (gray bars), and 1 μm Ang II (black bars). **E** Quantitative analysis of western blots for the secretion of CFD from CiGEnCs incubated in serum-free medium, 0.1 μM Ang II, and 1 μM Ang II for 0 h (white bars), 12 h (light gray bars), 24 h (dark gray bars), and 36 h (black bars). All data are the mean ± SD; *n* ≥ 3 per group; **p < 0.01 and ***p < 0.001 compared with secretion with serum-free medium; #*p* < 0.05 and ###*p* < 0.001 compared with incubation with 0.1 μM Ang II

CiGEnCs showed the ability to synthesize CFD, and the expression of CFD was upregulated in response to the enhancement of Ang II stimulation at both the mRNA (Fig. 3C) and protein (Fig. 3A, D) levels. The potential for CFD secretion by glomerular endothelial cells was investigated by treating CiGEnCs with Ang II at different time points and different concentrations. To exclude interference by the serum, CiGEnCs were incubated with serum-free medium, and CFD abundance in the cell culture supernatant obtained from plates with defined cell numbers was detected by western blotting. CFD secretion stimulated by Ang II from CiGEnCs was increased in a time-and dose-dependent manner (Fig. 3B, E).

### Local complement activation mediated by CiGEnC-derived CFD

**Fig. 4:**
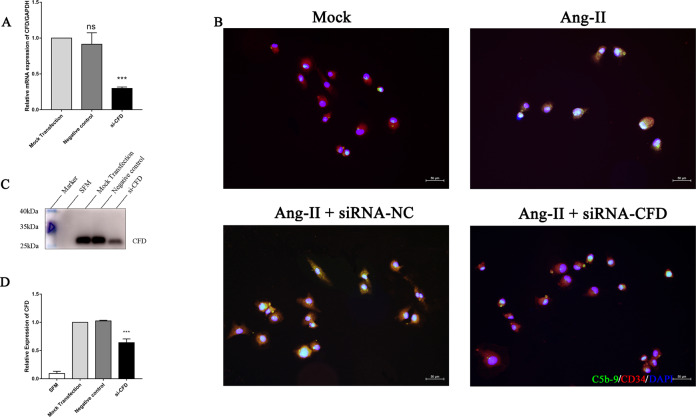
Reduction in local complement activation with gene silencing of CFD in CiGEnCs in vitro. **A** mRNA expression of CFD in CiGEnCs cultured for 24 h. **B** IF diagram showing the localization of C5b-9 (green), CD34 (red) and DAPI (blue). The merged image showing the colocalization of C5b-9 and CD34 (yellow). **C** Secretion of CFD in the supernatant of CiGEnCs cultured for 24 h. **D** Quantitative analysis of western blots for the secretion of CFD from CiGEnCs cultured for 24 h. SFM serum-free medium; All data are the mean ± SD; *n* ≥ 3 per group; ***p < 0.001 compared with the expression in the mock group. Scale bars represent 20 μm

To investigate the role of CFD secreted by CiGEnCs, we specifically interfered with CFD expression in CiGEnCs using siRNA against factor D (Si-CFD). The gene-silencing effect of siRNA treatment was examined at the mRNA and protein levels after transfection. After transfection with Si-CFD, CiGEnCs showed a significant decrease in the mRNA expression of CFD (Fig. 4A) and secretion of CFD into the supernatant (Fig. 4C, D). To investigate the role of CFD in local complement activation, the cells were incubated with serum from healthy humans to provide a complete complement system. We found C5b-9 (MAC) was highly enriched on the cell membrane of CiGEnCs after Ang II stimulation, as shown by co-staining with the endothelial cell marker CD34 (Fig. 4B). In addition, there was a significant reduction in the MAC on the cell membrane after interfering with the expression of CFD. The decrease in MAC deposition on the cell surface reflects a decline in the level of complement activation (Fig. 4B), which demonstrates a critical role of CFD expressed by CiGEnCs in local complement activation.

### Endothelial dysfunction mediated by CiGEnC-derived CFD in vitro

**Fig. 5:**
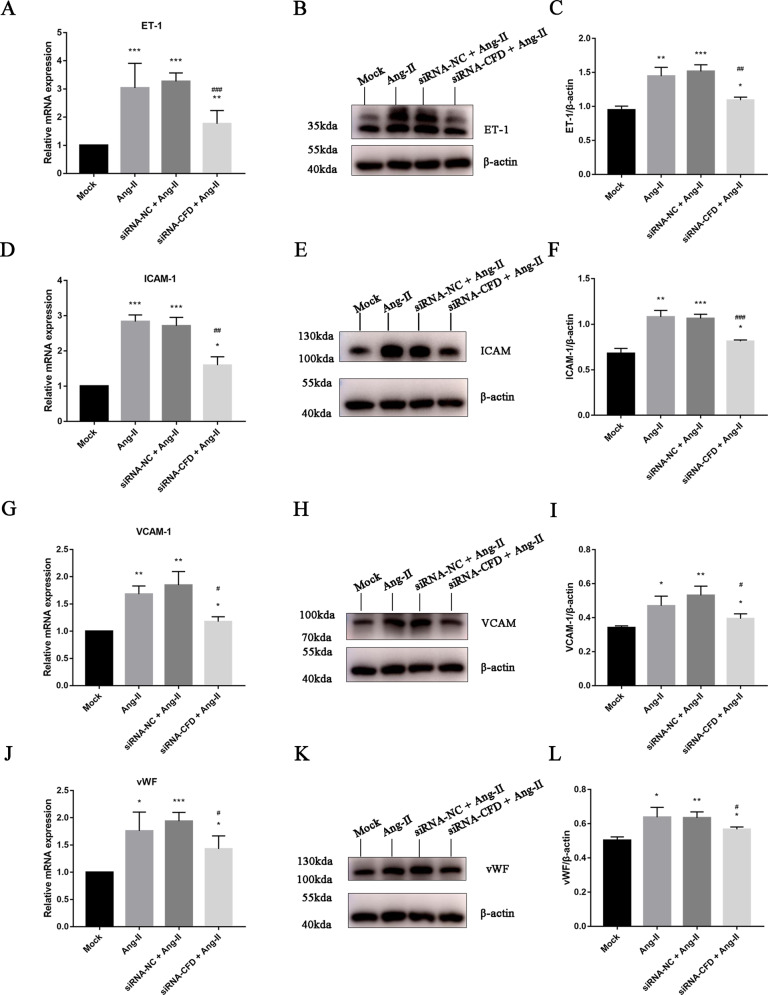
Attenuation of the Ang II-induced upregulation of ICAM-1, VCAM-1, vWF, and ET-1 expression in CiGEnCs with CFD knockdown. **A**, **D**, **G**, **J** Effects of CFD knockdown on the mRNA expression of ICAM-1, VCAM-1, vWF, and ET-1 in CiGEnCs. **B**, **E**, **H**, **K** Representative western blots showing changes in the protein levels of ICAM-1, VCAM-1, vWF, and ET-1 in CiGEnCs after CFD knockdown. **C**, **F**, **I**, **L** Protein levels of ICAM-1, VCAM-1, vWF, and ET-1 in CiGEnCs of each group shown as a ratio relative to β-actin. All data are the mean ± SD; *n* ≥ 3 per group; *p < 0.05, **p < 0.01, and ***p < 0.001 compared with the mock group; #*p* < 0.05, ##*p* < 0.01, and ###*p* < 0.001 compared with the group transfected with siRNA-negative control and stimulated with Ang II

The expression levels of ICAM-1, VCAM-1, vWF, and ET-1 in CiGEnCs were measured to evaluate the degree of endothelial dysfunction. The results showed that endothelial ICAM-1, VCAM-1, vWF, and ET-1 were expressed to a significantly lesser extent in the Si-CFD treatment group than in the negative control group under stimulation by Ang II (Fig. 5). Silencing the CFD gene in CiGEnCs attenuated Ang II-induced endothelial cell injury, indicating that CiGEnC-derived CFD may play a role in endothelial dysfunction.

### High expression of CFD in CiGEnCs

**Fig. 6:**
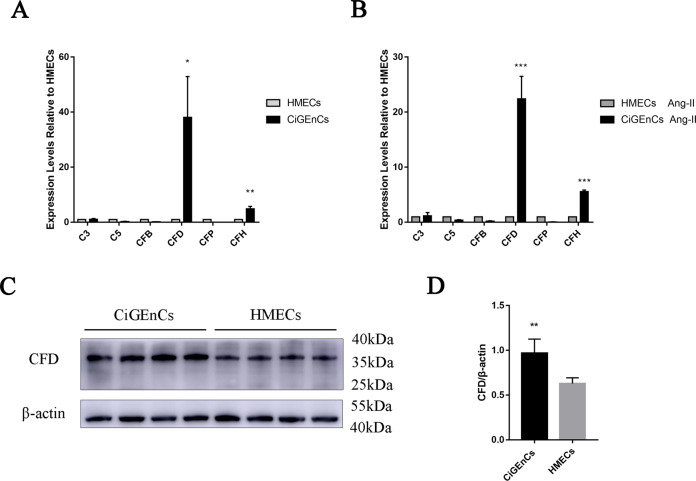
Differential expression of CFD in CiGEnCs and HMECs. **A** mRNA expression levels of complement AP components in conventionally cultured CiGEnCs and HMECs. **B** mRNA expression levels of complement AP components in CiGEnCs and HMECs stimulated with Ang II. **C**, **D** Protein expression levels of CFD in conventionally cultured CiGEnCs and HMECs. All data are the mean ± SD; *n* ≥ 3 per group; *p < 0.05, **p < 0.01, and ***p < 0.001 compared with HMECs

To investigate the unique role of glomerular endothelial cells in kidney injury, we compared the expression levels of complement proteins between CiGEnCs and HMECs. CFD expression in CiGEnCs was significantly higher than that in HMECs, both under conventional culture (Fig. 6A) and after stimulation by Ang II (Fig. 6B), and the difference in the mRNA expression of CFD was the most obvious compared with that of other various components in the AP. Furthermore, we examined cell lysates from two types of conventionally cultured cells by western blotting and found that the protein expression level of CFD in CiGEnCs was significantly higher than that in HMECs.

## Discussion

The pathogenesis of malignant nephrosclerosis is closely related to the abnormal activation of the complement AP. Recently, local activation of the AP in the kidneys, rather than activation in the systemic circulation, has been identified as a prominent factor in triggering malignant nephrosclerosis. The role of local complement activation in the kidneys has been confirmed in various renal diseases. As early as 20 years ago, Pratt et al found that C3, locally synthesized by renal tubular epithelial cells, plays an important pathophysiological role in renal transplant rejection [33]. Local complement synthesis has also been shown to mediate tissue damage in immune-related glomerulonephritis [34, 35]. In a mouse model of reperfusion renal injury, locally synthesized C3 but not C3 in circulation plays an important role [36]. In the past 10 years, research on extrahepatic complement synthesis has been focused on immune cells with little attention to renal intrinsic cells [17, 37, 38]. This study demonstrated the role of endothelial-derived CFD in malignant nephrosclerosis, contributing to a better understanding of local complement activation in the kidneys.

Local complement activation induced by CFD abnormalities has been shown to play a role in various diseases, such as age-related macular degeneration and systemic sclerosis [21, 23]. Characteristic vascular changes induced by local complement activation in the vascular bed in diabetic retinopathy and SARS-CoV2-induced complement-mediated endothelial damage are also related to CFD abnormalities [23, 24]. Notably, the level of CFD is the lowest among complement components in the blood (only 1–2 µg/mL), which is far lower than that of factor H (180–420 µg/mL), a complement regulatory protein [21, 39–41]. However, this study found a significant CFD deposits around the endothelial cells of patients with malignant nephrosclerosis, indicating that CFD plays an important role in malignant nephrosclerosis and suggesting that endothelial cells may be the main source of local renal CFD. Mechanistically, CFD, in response to Ang II stimulation, is over expressed in glomerular endothelial cells and secreted into the vascular lumen, where it interacts with circulating CFB and cleaves the latter to initiate the AP pathway, leading to sequential activation of C3b, C5 etc. and eventually MAC complex formation and deposition on endothelial cells.

We showed endothelial dysfunction in cultured CiGEnCs evidenced by enhanced transcription and translaton of ICAM-1, VCAM-1, vWF, and ET-1 in response to Ang II stimulation to simulate the activated renin-angiotensin system in patients with malignant HTN. ICAM-1 and VCAM-1 are members of the immunoglobulin superfamily, and their increased expression indicates that endothelial cells lose their antithrombotic properties and become more reactive with leukocytes [26–28]. The abnormal elevation of VWF, a glycoprotein mainly synthesized by endothelial cells and involved in arterial thrombosis, suggests a functional disturbance in the ability of endothelial cells to participate in coagulation and fibrinolysis [29, 30]. ET-1 is a potent vasoconstrictor with pro-regenerative, pro-fibrotic, and pro-inflammatory properties and is a key player in endothelial cell dysfunction [31, 32]. Endothelial cells play an important role in the pathogenesis of various kidney diseases by regulating the exudation of immune cells and vascular permeability, modulating anti-thrombotic and anti-inflammatory effects, and maintaining tissue perfusion and vascular tension [42–46]. Our results demonstrated that endothelial cells actively participated in the progression of malignant nephrosclerosis by expressing CFD. Endothelial cells specifically differentiated from different organs and tissues are heterogeneous in structure and function [47]. The kidneys are one of the main sources of extrahepatic complement components [48]. Complement expression in glomerular endothelial cells is higher than that in other types of endothelial cells [49]. Our results showed that the expression of the CFD in renal endothelial cells was significantly higher than that in dermal microvascular endothelial cells, which allowed glomerular endothelial cells to participate in local complement activation in the kidneys.

In conclusion, our study showed that local renal complement activation via overexpression and secretion of CFD by glomerular endothelial cells is an important pathway of complement activation in malignant nephrosclerosis. Endothelial cells are not only passive victims but also active participants and contributors to malignant nephrosclerosis. Endothelial-derived CFD initiates and promotes vascular endothelial injury by activating the complement system, which plays an important role in the pathogenesis of malignant nephrosclerosis.

## Supplementary information

Supplementary Table 1

Supplementary Table 2
