# Supplementary material for: Endothelial-derived complement factor D contributes to endothelial dysfunction in malignant nephrosclerosis via local complement activation
Source: Hypertens Res. 2023 May 15;46(7):1759–70. doi: 10.1038/s41440-023-01300-3 (PMC10184087; doi:10.1038/s41440-023-01300-3)
Supplement: Supplementary file 1 — Supplementary Table 1 [file 41440_2023_1300_MOESM1_ESM.docx]

| **Supplementary Table 1: Demographic and clinical data of patients with malignant  nephrosclerosis and normal controls** | | | |
| --- | --- | --- | --- |
| Characteristics | Malignant nephrosclerosis (n=5) | Normal controls (n =3) | P-value |
| Age (years), mean±SD | 38.60±11.63 | 45.00±4.58 | 0.408 |
| Gender(male/femsle), n/n | 3/2 | 1/2 | 0.465 |
| History of hypertension, n(%) | 5(100) | 0 (0) | 0.005 |
| SBP(mmHg), mean±SD | 191.20±10.13 | 122.00±2.65 | ＜0.001 |
| DBP(mmHg), mean±SD | 117.20±15.06 | 79.67±3.21 | 0.006 |
| SCr(μmol/L), median(IQR) | 421.00±227.43 | 56.00±8.72 | 0.036 |
| eGFR(ml/min/1.73m^2^), mean±SD | 20.16±12.44 | 111.53±9.22 | ＜0.001 |
| Proteinuria(g/24h), median(IQR) | 2.32±1.92 | 0.06±0.03 | 0.095 |
| Haematuria, n(%) | 5(100) | 0(0) | 0.005 |
| Haemoglobin(g/L), mean±SD | 107.50±27.46 | 137.00±16.52 | 0.148 |
| Platelets(×10^9^/L), mean±SD | 172.40±87.02 | 219.33±48.35 | 0.432 |
| Serum C3(g/L), mean±SD | 1.13±0.19 | 1.17±0.13 | 0.753 |
| Serum C4(g/L), mean±SD | 0.35±0.05 | 0.35±0.04 | 0.917 |
