# Supplementary material for: Endothelial-derived complement factor D contributes to endothelial dysfunction in malignant nephrosclerosis via local complement activation
Source: Hypertens Res. 2023 May 15;46(7):1759–70. doi: 10.1038/s41440-023-01300-3 (PMC10184087; doi:10.1038/s41440-023-01300-3)
Supplement: Supplementary file 2 — Supplementary Table 2 [file 41440_2023_1300_MOESM2_ESM.docx]

| **Supplementary Table 2: The primer sequences used in this study** | | |
| --- | --- | --- |
| Target ID | Forward primer | Reverse primer |
| GAPDH | ACAACTTTGGTATCGTGGAAGG | GCCATCACGCCACAGTTTC |
| FD | GCAAGAAGCCCGGGATCTAC | GTTGCTTGGGTGACCCTGAC |
| FB | CAGCCCTGGAAGTCAAGAGAACAC | CCGCAGAAGCAGCATCTTACCTAC |
| FH | GTGACTTACACTTGTGCAACAT | GGGCTCCTACATTGATAACGTA |
| FP | TATGAAGAATCCTCCGGCAAG | ACTACGTTTCTGGTAGGCAAAG |
| C3 | TACGGTGGTGGCTATGGCTCTAC | CTGGCTCGTGGGAATGAAGAACTG |
| C5 | GGGCTGGCTGGTCTAGGAAGG | GCTGGATGATGAGAGGCACACAC |
| vWF | CCTGTTACTATGACGGTGAGAT | CATGAAGCCATCCTCACAGTAG |
| ICAM-1 | TGCAAGAAGATAGCCAACCAAT | GTACACGGTGAGGAAGGTTTTA |
| VCAM-1 | CAGGCTGGAGATAGACTTACTG | CCTCAATGACAGGAGTAAAGGT |
| ET-1 | TAGCCAAAAAGACAAGAAGTGC | TTCTTCCTCTCACTAACTGCTG |
